# Behavioural and Physiological Correlates of the Canine Frustration Questionnaire

**DOI:** 10.3390/ani11123346

**Published:** 2021-11-23

**Authors:** Kevin J. McPeake, Lisa M. Collins, Helen Zulch, Daniel S. Mills

**Affiliations:** 1 The Royal (Dick) School of Veterinary Studies, Easter Bush Campus, Midlothian EH25 9RG, UK; 2 Animal Behaviour Cognition and Welfare Group, School of Life Sciences, University of Lincoln, Lincoln LN6 7TS, UK; hzulch@lincoln.ac.uk (H.Z.); dmills@lincoln.ac.uk (D.S.M.); 3 Faculty of Biological Sciences, University of Leeds, Leeds LS2 9JT, UK; l.collins@leeds.ac.uk

**Keywords:** canine behaviour, frustration, psychometrics, veterinary behaviour, behavioural assessment

## Abstract

**Simple Summary:**

Frustration is a negative emotional state implicated in a range of canine behaviour problems. The Canine Frustration Questionnaire (CFQ) is an owner questionnaire developed to measure frustration tendencies in dogs. This study looks at behavioural and physiological measures and their relationship with the CFQ. A series of tests were designed to induce frustration in dogs, and these were completed by 44 dogs; each dog owner completed a CFQ. Specific behavioural measures were coded from the test footage, and the relationships with the CFQ scores were assessed. In addition, a saliva sample was collected before and after the test in 39 dogs so that cortisol, a measure of physiological arousal, could be measured. Various behavioural test measures (e.g., vocalising and lunging) were associated with CFQ scores. Cortisol change and cortisol levels after the tests were greater in dogs who were more highly frustrated. These results support the use of owner report through the CFQ to measure frustration tendencies.

**Abstract:**

Frustration is a negative emotional state implicated in a range of canine behaviour problems. The Canine Frustration Questionnaire (CFQ) is the first psychometric tool developed to assess frustration tendencies in dogs based on owner report. However, to date, no published studies have assessed behavioural and physiological correlates of this trait. A novel behaviour test battery was developed to induce frustration in dogs, mapping onto the CFQ. Forty-four dogs were recruited and filmed whilst undertaking the test battery, and a CFQ was completed by each owner. Targeted behavioural measures were assessed from this footage, based on hypotheses aimed at evaluating convergent and discriminant validity with facets of the CFQ. In addition, a saliva sample was collected pre- and post-testing for 39 dogs, and a cortisol assay performed using ELISA to provide a physiological measure of arousal. A range of predicted behavioural test measures (e.g., vocalising and lunging) positively correlated with CFQ scores. For 22 dogs with pre-test salivary cortisol levels of <4 ng/mL (indicative of normal arousal at baseline), cortisol change and post-test cortisol levels positively correlated with the CFQ PC5 ‘Frustration coping’ score. These results provide further evidence of the validity of frustration tendencies as measured by owner report through the CFQ.

## 1. Introduction

Frustration is an emotional response to the violation of a given expectation [1], which may involve engagement of reactive aggression (RAGE) [2]. Frustration can arise in a variety of situations, including absent, reduced, or delayed rewards [3], where one is thwarted from achieving a goal [4,5] as well as in circumstances where there is a perceived loss of autonomy [6].

Frustration-related behaviours can vary depending on the context, and can include increased efforts to achieve a desired goal [6] and vocalisations [7]. In the field of clinical animal behaviour, frustration has been considered an important negative emotion implicated in a range of common behaviour problems in dogs. Barrier frustration can arise where a dog is thwarted from obtaining a desired stimulus e.g., by a door or leash restraint [6], and can result in redirected [8] and/or aggressive behaviours [9]. Frustration has been highlighted as a key emotional differential within the umbrella term of “separation-related problems” in dogs [10,11,12,13]. Links have been described between frustration and the development of repetitive and compulsive behaviour problems [14,15]. Indeed, a lack of autonomous control over the environment occurs in all of these contexts, and is a contextual hallmark for frustration [2].

In the human literature, attention has been given to the study of frustration tendencies as a trait [16,17,18]. However, in contrast, most studies of frustration in dogs have focused on it as an emotional reaction, typically induced through reinforcement omission and extinction protocols in experimental settings [7,19,20]. A notable exception is the development of the Canine Frustration Questionnaire (CFQ), a psychometric tool for the quantification of frustration tendencies in dogs. The CFQ assesses various manifestations of frustration across common contexts in owned dogs. The CFQ has been shown to be reliable at intra- and inter-rater levels, and has face validity including elements of convergent validity (see McPeake, et al. [21] for further details).

A variety of methods have been used to provide behavioural validation of canine psychometric tools. Concurrent validity is where a test correlates highly with another valid test measuring the same construct [22], and has been demonstrated for the Dog Impulsivity Assessment Scale (DIAS), using measures from a laboratory-based delayed reward paradigm [23]. Concurrent validity has also been shown between different questionnaires (the refined Monash Canine Personality Questionnaire [24] and the Dog Personality Questionnaire [25]) that appear to assess the same personality trait [26]. For the validation of any personality or temperament trait scale, the use of a behaviour test battery is preferable, since it has the potential to demonstrate objective validity, as seen by the replication of behaviours across a range of contexts, which is consistent with the characteristics of a psychological trait.

Physiological measures may also form an important part of psychometric validation [22]. The use of salivary cortisol as a potential correlate of interest has yielded mixed results. Dreschel and Granger [27] found that salivary cortisol levels increased following exposure to thunderstorm noise, but they did not correlate with relevant measures from the C-BARQ. However, the time elapsed post-test before collection may be important. Indeed, Lensen, et al. [28] found that samples collected 10 min post-test were associated with desirable behaviours as measured by the C-BARQ, whilst samples collected 40 min post-test were associated with non-desirable behaviour.

The primary aim of this study was to demonstrate convergent validity between the CFQ and selected behavioural and physiological measures. This adds value to the current literature on canine frustration through increasing confidence in using the CFQ for the objective quantification of frustration tendencies through owner report.

Most important in the validation process of trait level frustration were the hypotheses that the CFQ Overall Questionnaire Score (OQS) as well as the principal component believed to represent ‘general frustration’ (PC1) [21], would positively correlate with cumulative measures of vocalisation across all tests in the battery. Vocalisation was selected as the primary behavioural test measure as it could be displayed in all behaviour tests, and is commonly reported as a sign of frustration in dogs in experimental settings [7].

Secondary hypotheses were also generated for other facets of frustration defined by the remaining PCs of the CFQ. It was predicted that positive correlations would be found between:

- PC2 (‘Barrier frustration/perseverance’) and lunging (since frustration-related lunging is relevant once the animal has identified that there is a barrier) and potentially vocalising when dogs were unable to access certain items;
- PC3 (‘Unmet expectations’) and vocalisation when there was a delay to the dog being able to leave the room, and potentially ambulation when the dog was ignored, as a sign of restlessness relating to frustration [7];
- PC4 (‘Autonomous control’) and the frequency of aggressive behaviours when items were removed from the dog as the animal sought to control the resource, as well as the frequency of pawing/scratching at a barrier separating the dog from an experimenter, as more frustrated dogs would be expected to try to remove the barrier themselves;
- PC5 (‘Frustration coping’) and both lunging and vocalisation when dogs were unable to access food.

It was also hypothesised that the correlation between each of these behavioural measures and the related CFQ PC should also be higher than its correlation with other CFQ PCs.

From a physiological perspective, it was hypothesised that changes in salivary cortisol after the completion of testing would be greater in those dogs experiencing greater arousal during frustration, as assessed by CFQ OQS/PC scores and their behavioural test correlates.

## 2. Materials and Methods

### 2.1. Subjects

Dogs were recruited in May 2019 through opportunistic sampling of willing owners registered on the University of Lincoln PetsCanDo database (http://www.lincolnpetscando.co.uk/, accessed on: 12 April 2019), and through known staff/student contacts. Initial criteria for inclusion were that the owner could bring their dog for a single visit to the University of Lincoln, and that there was no restriction on their dog eating around 40–50 small pieces of food to be used during the testing session. Pork cocktail sausage (each piece approximately 0.8 g) were used as food treats, unless the dog had dietary sensitivities, in which case the owner was asked to provide an equivalent number of suitable food treats which the dog would readily consume. Initial exclusion criteria included any known sensory deficit relating to sight or hearing, or the presence of any medical problem which precluded the use of a flat collar/harness and lead. A total of 46 dogs were recruited for the study, with two dogs subsequently excluded due to a failure to habituate to the environment and refusal to consume food in the absence of their owners. The 44 dogs tested were aged from 11 months to 14 years old (average 6 years 0 months). The majority of dogs were neutered: male neutered (*n* = 14, 31.8%, including one ‘chemically castrated’ with a hormonal implant), female neutered (*n* = 17, 38.6%), male entire (*n* = 7, 15.9%), female entire (*n* = 6, 13.6%). Bodyweight of dogs ranged from 7 kg to 40 kg (average 19.5 kg). Most dogs were classed as pure bred (*n* = 37, 84.0%), with 7 cross bred dogs (16.0%). Pure bred dogs were comprised of 14 breeds: Australian Kelpie (1), Belgian Malinois (1), Border Collie (2), Border Terrier (7), Cavalier King Charles Spaniel (2), Cocker Spaniel (3), English Springer Spaniel (3), French Bulldog (1), Golden Retriever (1), Hungarian Vizsla (1), Labrador Retriever (12), Large Münsterländer (1), Nova Scotia Duck Tolling Retriever (1), and Shetland Sheepdog (1).

Current medical problems were reported in 12 dogs (seven with orthopaedic problems; four with dermatological problems; one with atypical hypoadrenocorticism), which were all receiving treatment ranging from supplements to prescription medications. Dietary sensitivities were reported in five dogs, which precluded the use of pork cocktail sausages. Current behaviour problems were reported in three dogs, and each of these was receiving psychoactive medication (imepitoin for anxiety and noise sensitivity (1); fluoxetine and imepitoin for high impulsivity and anxiety (1); clomipramine for anxiety when on walks and around traffic (1)). Both behaviour test and CFQ score data were visualised from subjects with these reported medical and behavioural problems, in order to evaluate if they were outliers, with a view to their exclusion.

#### Assessment of Frustration Tendencies Using the Canine Frustration Questionnaire

Owners were asked to complete the 21 item Canine Frustration Questionnaire (CFQ) for their dog, and scores were calculated as per McPeake et al. [21], generating a single ‘overall questionnaire score’ (OQS) and five principal component (PC1-5) scores for each dog, each score within a range of 0.2–1.0.

### 2.2. Behaviour Test Battery

All tests were developed so that they could be performed in a test room under controlled conditions with a single experimenter and without the need for pre-training. Given the potential for lunging behaviour, dogs were either fitted with a flat, non-tightening buckle collar (Halti^®^ Collar, Company of Animals, Surrey, UK), or their own flat collar/harness, if their owner preferred. An elastic bungee lead (Halti^®^ All-in-one Lead, Company of Animals, Surrey, UK) was used to provide some shock absorbance and reduce the potential for discomfort during lunging whilst on lead during the tests.

Existing behaviour tests purportedly inducing frustration in dogs were identified from relevant literature, and assessed for inclusion/adaptation. Details of the tests can be found in Table 1, with their potential association with different elements of frustration as assessed by CFQ (described in Supplementary Materials Table S1–S5). These are: Test 1—Downshifting; Test 2—Inability to access items; Test 3—Delayed leaving; Test 4—Dog ignored; Test 5—Access to food denied, tethered; and Test 6—Dog left alone. A pilot study of the test battery was performed with two dogs, and no revisions were made. Full details of the test protocols can be found in Supplementary Material Figure S1, with sample videos showing their execution in practice in Supplementary Material Video S1.

### 2.3. Salivary Cortisol

Saliva collection was attempted in all dogs. Pre-test saliva samples were taken after habituation, immediately before commencing the battery of behaviour tests; post-test saliva samples were collected 2 min after the end of the test battery (as per the protocol of de Carvalho, et al. [30]). Saliva was collected using sterile flocked nylon swabs (Thermo Scientific™ Sterilin™ flocked plain swab, Thermo Scientific, Cambridge, UK; Cat.no. 11399173). Food treats were held in the experimenter’s left hand to encourage the dog to approach/sniff the hand and to stimulate saliva production. A swab was gently inserted into the buccal pouch and rolled around for approximately 5–10 s, or until saliva was evident on the swab tip. The swab was instantly re-sheathed and kept on an ice pack. Within 1 h, swab tips were then cut off into Eppendorf tubes and then frozen at −20 °C, before being transferred to a freezer maintained at −80 °C within 24 h for storage. All sample storage and laboratory analyses were performed at Joseph Banks Laboratories, University of Lincoln in January 2020.

Samples were thawed at room temperature, and centrifuged for 10 s at 6000× *g*. A sterile pipette was used to harvest the pooled saliva from the bottom of the Eppendorf tube. Analysis of cortisol was performed using a species independent cortisol enzyme-linked immunosorbent assay (ELISA) kit (DetectX^®^, Arbor Assays, Ann Arbor, MI, USA, Cat. No. K003-H1W/X012-1EA). The product protocol for analysing salivary cortisol from the samples was followed (https://www.arborassays.com/documentation/inserts/K003-H.pdf, accessed on: 7 January 2020). A plate reader (Bio-Rad iMark^TM^ Microplate reader, software MPM 6) was used at 450 nm, as per the ELISA kit instructions, to generate the results.

### 2.4. Test Room Set-Up

**Figure 1:**
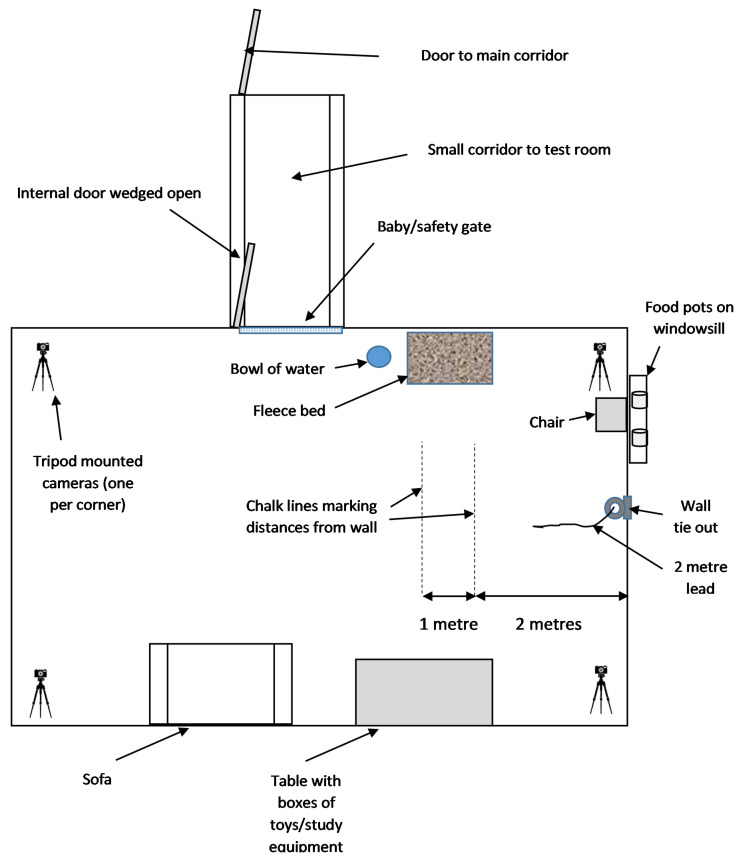
Layout of test room for behavioural tests (sizes are approximate and not to scale).

All testing was conducted within the Animal Behaviour Clinic, University of Lincoln (https://animalbehaviourclinic.lincoln.ac.uk/, accessed on: 7 April 2019). The test room measured 7.35 × 5.15 metres, with permanent non-slip rubber flooring throughout, and had in place a metal wall tie with a 2-metre long bungee lead attached. The test room was separated from a short corridor by a 1-metre-tall metal safety/baby gate, with a solid door leading to the main corridor. Air conditioning was set to maintain air temperature at 21 °C throughout all testing. A thick fleece blanket and bowl of water were provided within the room for the dog to use if so desired. The test room set up is shown in Figure 1.

All testing was filmed using four GoPro™ Hero5Session cameras (product number: CHDHS-502; 4 K resolution, 30 frames per second). Cameras were mounted on tripods, with one positioned in each corner of the room to capture video and audio of the testing. A GoPro™ Wi-Fi Smart Remote Control (product number: GP2039) was used to synchronise the start of recording of all four cameras.

### 2.5. Test Schedule

After arrival in the test room, each dog was given around 10 min to habituate, where they could explore the room off lead. During testing, after each short test, the dog was permitted access to what was being denied. Regular 2-min breaks were included, which took place in the test room between tests, with a longer toilet break outside of the test room approximately half-way through the test battery. Overall, the full test schedule took approximately 37 min including habituation and breaks. Strategies were determined a priori to deal with any aggressive behaviours or excessive frustration arising during testing. Additionally, a protocol was developed for the end of each test to ensure that undesirable behaviours (e.g., jumping up or vocalising) were not reinforced, the experimenter would wait for, or request, a more appropriate behaviour before allowing the dog to access what it could not previously obtain. A detailed guide to each test, including these strategies, is provided in Supplementary Material Figure S1.

The test order was counterbalanced so that every other dog undertook the tests either from 1 to 6, or from 6 to 1 (see Supplementary Material Tables S6 and S7).

### 2.6. Ethogram

An ethogram detailing the behavioural measures and corresponding tests selected for correlational analysis with CFQ is shown in Table 2.

### 2.7. Coding of Video Footage

Video footage from each camera was transferred and saved in a file linked to the subject’s assigned code. The software Solomon Coder© 2019 (Péter; Version: beta 19.08.02) was used as a platform for manual key-press coding of the selected behavioural variables. The primary author (KM) performed all coding, and results for each dog were subsequently saved in Microsoft Excel files.

### 2.8. Inter-Rater Reliability

In order to test for inter-rater reliability, double coding of 11 randomly selected dogs (25%) was undertaken by a second coder with a post-graduate qualification in Clinical Animal Behaviour and previous experience of coding behaviour using the same software.

### 2.9. Statistical Analysis

All statistical analyses were conducted using IBM SPSS Version 25. Visual assessment of histograms of CFQ OQS/PC scores in the population of 44 dogs tested together with Kolmogorov–Smirnov tests revealed that OQS, PC2, PC3, and PC4 scores were normally distributed (*p* > 0.05), whereas PC1 and PC5 were not normally distributed (*p* < 0.05). Non-parametric tests were therefore used for statistical analysis. Values for all subsequent tests were deemed significant at the *p* < 0.05 level, without correction for multiple testing given the a priori rationale for the hypotheses being tested. For all correlational analysis, values of 0.5–1.0 were deemed ‘strong’, 0.3–0.49 deemed ‘moderate’, and <0.299 weak.

Inter-rater reliability assessment (IRR) was assessed using a two-way mixed, absolute agreement, average-measures intraclass correlation coefficient (ICC) for the seven behavioural measures. Mann–Whitney tests were used to compare the data of the two populations used in the experimental counterbalance.

To test the hypothesis that CFQ OQS and PC1 (General frustration) would be associated with cumulative measures of vocalising—the number of tests where vocalising (barking/whining) occurred, and, the total frequency of vocalising (barking/whining)—across all tests in the battery, Spearman’s correlation coefficient was used.

For the secondary hypotheses, correlational analyses were initially performed with the selected behavioural measures of single tests and the corresponding element of CFQ (i.e., PC2-5). Where associations were established, further correlational analyses of the behavioural measure versus the other PCs (PC2-5) were undertaken to examine the hypothesis concerning the strength of association with the primary PC of interest.

Mann–Whitney tests were used to establish whether the distribution of CFQ scores obtained from the population of dogs used in testing was significantly different to the population of dogs used in the development of the original questionnaire.

To test the hypothesis that changes in salivary cortisol during the test battery would be associated with CFQ OQS/PC scores and vocalising measures, Spearman’s correlation coefficient was used. For salivary cortisol levels in dogs, 4 ng/mL has been proposed as a threshold for ‘stress’ [30,31]. Test subjects with pre-test cortisol levels of >4 ng/mL were removed, as they represented individuals who were already highly physiologically aroused at the onset of the test; measuring the change in salivary cortisol in this subgroup over the course of test battery may undermine the test hypothesis. Associations were also tested between CFQ OQS/PC scores and absolute salivary cortisol levels, both pre-test and post-test. Mann–Whitney tests were used to test for differences in CFQ OQS/PCs in dogs grouped by post-test cortisol levels less or greater than 4 ng/mL.

## 3. Results

### 3.1. Inter-Rater Reliability and Testing for Order Effects

Intraclass correlation coefficients (ICC) were very high in all cases (>0.928; average 0.987). Full details for the ICC for each behavioural measure can be found in Supplementary Material Table S8. CFQ OQS and PC1-5 scores for the two groups used in the counterbalancing test order did not significantly differ, nor was there a difference between the two subgroups in the frequency of key behavioural measures (Supplementary Material Tables S9 and S10).

### 3.2. Relationship between Vocalising and CFQ OQS/PC1 (General Frustration) Scores

There was a moderate positive correlation between the CFQ OQS and total number of tests in which vocalising occurred (*r_s_* = 0.383, *n* = 39, *p* = 0.016), as well as the total frequency of vocalisation across all tests (*r_s_* = 0.339, *n* = 39, *p* = 0.035). There were no significant correlations between these behavioural measures and CFQ PC1 (*r_s_* = 0.203, *p* = 0.216, and *r_s_* = 0.204, *p* = 0.524) respectively.

### 3.3. Relationship between Single Test Measures and CFQ PC2-5

In relation to barrier frustration (PC2) and the total frequency of lunging in Test 2 (inability to access items), there was no significant correlation when including all dogs (*r_s_* = 0.235, *p* = 0.134, *n* = 44). However, on visual inspection of the scatterplot, it was decided to test for an association between PC2 and only those dogs who lunged greater than twice (i.e., 3 and above) during the test. This revealed a moderate positive correlation (*r_s_* = 0.431, *p* < 0.017, *n* = 30). There was no significant correlation between PC2 and vocalising in test 2 (*r_s_* = 0.064, *p* = 0.685).

For unmet expectations (PC3), the frequency of vocalising in Test 3 (delayed leaving) significantly positively correlated with the score on this PC (*r_s_* = 0.313, all *p* = 0.038). There was no significant correlation between PC3 score and duration of ambulation in Test 4 (dog ignored) (*r_s_* = 0.146, *p* = 0.344).

There was a significant positive correlation (*r_s_* = 0.376, *p* = 0.012) between PC4 (Autonomous control) scores and frequency of pawing/scratching at the barrier when separated from the experimenter in Tests 6a/6b (dog left alone). No aggressive behaviours were displayed in test 2b (removal of items following test 2a), so no analysis was performed.

There was a significant positive correlation (*r_s_* = 0.447, *p* = 0.003) between PC5 (Frustration coping) and the frequency of vocalisation in Test 5 (access to food denied, tethered). There was no significant correlation between PC5 and frequency of lunging in Test 5 (*r_s_* = 0.074, *p* = 0.639), and the scatterplot did not suggest an association between PC5 and those dogs who lunged greater than twice, unlike the PC2/Test 2 (inability to access items) relationship above.

Generally, the correlation was higher for the significant correlations described above compared to the behavioural measure and other PCs (see Supplementary Material Table S11), with the exception of the frequency of vocalising in Test 3 (delayed leaving) and PC3, which correlated more highly with PC5. This latter relationship was predicted (see Supplementary Materials Table S5).

### 3.4. Comparison of CFQ Scores in Behaviour Test Dogs and the Wider Population

There was no significant differences in the CFQ OQS, nor PC1, PC4, or PC5 scores of the test population and the population used in the development of CFQ (*n* = 2346; [21]. However, the behaviour test group scored significantly higher for PC2 (barrier frustration) and PC3 (unmet expectations) (*p*’s = 0.041 and 0.003, respectively) (see Supplementary Material Table S12 for full results).

### 3.5. Salivary Cortisol

Paired saliva samples were successfully collected from 39 dogs. Pre-test cortisol levels exceeded the 4 ng/mL threshold for 17 dogs. For the remaining 22 dogs, median salivary cortisol levels pre-test were 2.9 ng/mL (range 0.20 to 3.81 ng/mL) and post-test were 3.04 ng/mL (range 0.90 to 6.90), with a pre- to post test-change of 0.43 ng/mL (range −2.59 to +6.70 ng/mL).

**Figure 2:**
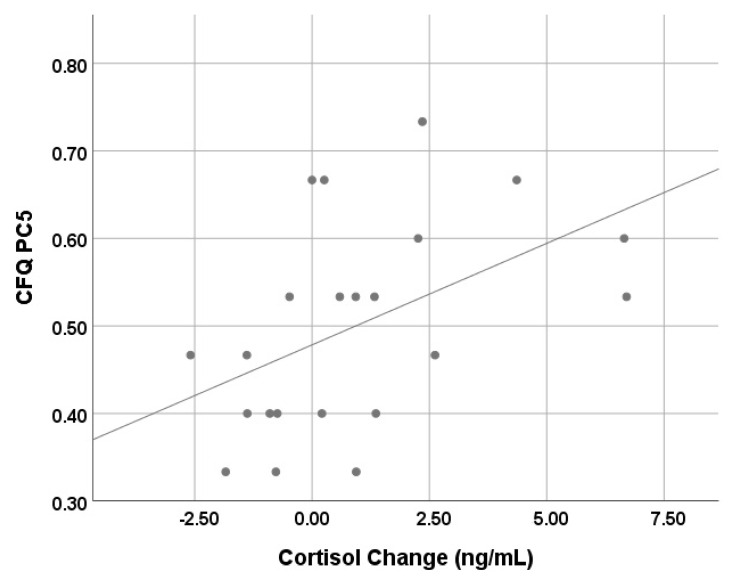
Scatter plot of CFQ PC5 (‘Frustration coping’) score versus pre-test to post-test cortisol change (ng/mL).

There was a significant positive correlation between CFQ PC5 (‘Frustration coping’) and pre-test to post-test change in cortisol (*r_s_* = 0.525, *p* = 0.012; Figure 2). Additionally, there was an association between CFQ PC5 (‘Frustration coping’) and post-test cortisol (*r_s_* = 0.477, *p* = 0.025). There were no significant associations between the other measures (full data in Supplementary Material Table S13).

Median scores for CFQ OQS/all PCs, as well as vocalising measures, were numerically greater in the group of dogs with post-test cortisol >4.0 ng/mL (*n* = 7) than those with levels <4.0 ng/mL (*n* = 15) (Table 3). Mann–Whitney tests revealed that this difference was statistically significant for CFQ PC5, and total number of tests where vocalising was observed.

## 4. Discussion

The primary aim of this study was to demonstrate convergent validity between the CFQ and selected behavioural and physiological measures. Most importantly, trait level frustration (CFQ OQS), with the validity of CFQ PC scores examined in the context of other specific behaviours linked to the behaviour test battery. Critical to this is the quality of the behaviour measures. Although a new behaviour test protocol was developed and the test battery in itself had not been previously validated, the tests involved were formulated either from already published tests [7,19,20,29], or created based on expert opinion concerning the expression of frustration, and therefore had at least face validity. Inter-rater reliability (IRR) [32,33] for the measures chosen, which were selected because of their ease of identification, was ‘excellent’, reinforcing the quality of the behaviour measures. Aggression was not observed in our subjects, and this may reflect the desire for the tests not to be overly taxing for dogs, or, reflects the nature of the subjects chosen. The former may be more likely, since the dogs in the sample were broadly representative of the wider population, and if anything scored higher on certain elements of CFQ (barrier frustration and unmet expectations) compared with the previous McPeake et al. [21] paper. Thus, the behaviour tests were probably a relatively low stress challenge that can be easily used in the field. It is also worth noting that aggressive behaviour in frustrating contexts, such as around food, has been shown to have questionable reliability in other contexts [34], so the absence of aggressive behaviour in the test battery may not preclude the exhibition of such behaviour in a more natural context. This, together with recent work by Clay, et al. [35], emphasises the value of a psychobiological approach, focusing on evaluating traits such as frustration, fear, and sociability which underlie specific problem behaviours rather than single outcomes, such as food guarding, which may be triggered by diverse stimuli [36]. Owner reported frequency of clearly defined incidences of ‘resource guarding’ in the home, such as growling, snapping, and biting around food and toys, may be a better potential correlate for examining convergent validity with frustration scores.

**Figure 3:**
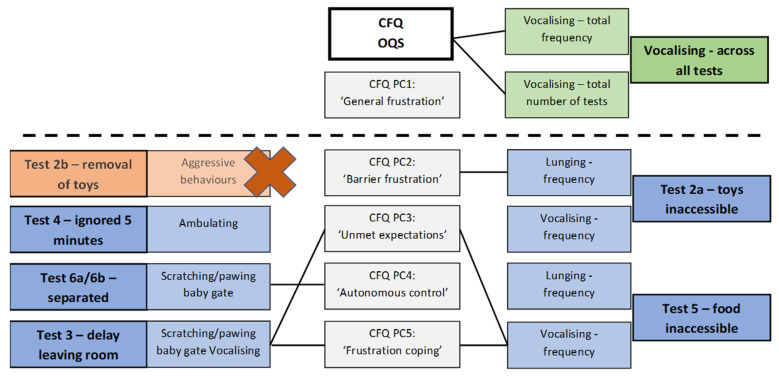
Illustration of relationship between Canine Frustration Questionnaire (CFQ) OQS (overall questionnaire score) and PC (principal component) score with selected behaviour test battery measures. Above the dotted line represents CFQ OQS/PC1 testing with behaviour measures from full test battery (represented by green box); below the dotted line represents CFQ PC2-PC5 testing with single behaviour test measures (represented by blue box) The solid line represents a significant positive correlation. Red cross indicates absence of behaviour seen during test therefore no associations tested, and orange box represents proposed behavioural measure not tested.

The main relationships between CFQ OQS/PCs and behavioural measures are shown in Figure 3. With the exception of PC1 (‘General frustration’), at least one expected behavioural correlate was found for each component of the CFQ, supporting the validity of the questionnaire and its principal components. Vocalising has been highlighted as a common sign of frustration in dogs [7,19,20], and so was selected as the key indicator of frustration. Convergent validity was demonstrated between CFQ OQS and both the frequency of vocalising across all tests, as well as the number of tests where vocalising occurred, i.e., dogs with higher CFQ OQS vocalise more frequently, and vocalise in more tests of frustration within the test battery created. However, we did not show its predicted association with PC1 (‘General frustration’). In hindsight, this is perhaps not surprising. Although PC1 is described as reflecting ‘general frustration’, the strongest loading items in the CFQ relate to the frequency of frustration in particular contexts, rather than the intensity of the response (i.e., “my dog becomes frustrated in a large range of situations”; “there are days when my dog seems to become more easily frustrated than others for no apparent reason”; “my dog appears to become frustrated frequently (e.g., at least once daily)”). Such items are less amenable to study from a test battery administered on a single day. Alternative means for assessing the validity of CFQ PC1 scores could include their interpretation alongside owner-kept diaries of frequency and range of situations in which frustration-related problems occur, and exploration of fluctuations within these reports. Other behaviours, such as yawning, lip licking, ear posture, and other facial actions, have been used to assess frustration [7,19,20,37,38], but these measures were not used in this study because of the challenge of capturing them reliably.

Considering the secondary hypotheses on the relationship between the PC scores and behaviour, at least one independent behaviour measure concurred with the score of the related PCs. It seems reasonable to conclude that PCs within the CFQ are not only robust, but also reflect real world behaviour changes in the context of frustration. The results for PC5, ‘frustration coping’, were further reinforced by their correlation with salivary cortisol change over the test battery. When considering absolute cortisol levels, whilst there were no associations with pre-test cortisol and CFQ or behavioural test measures (as expected), there was a positive association with post-test cortisol and PC5, ‘frustration coping’ (Table S13). This is consistent with cortisol levels reflecting the ability to cope with frustration, with absolute post-test levels >4 ng/mL indicating increased arousal/“stress” levels [30,31]. This suggestion is reinforced by the finding that PC5, ‘frustration coping’ scores and the total number of behaviour tests where vocalising occurred was significantly higher for those dogs with absolute post-test cortisol levels >4 ng/mL, compared to those where post-test cortisol remained below this threshold.

A behavioural point of note is one of the parameters used to assess lunging behaviour i.e., greater than 2 lunges. In a novel situation, the motivation to lunge will be affected by both the salience of any potential resource to which the animal may wish to gain access, and the dog’s obedience training. Thus, a single lunge may be an insensitive measure of frustration; only those subjects lunging on multiple occasions are expressing a quantifiable response to the consequences of a barrier which has been found (through the first lunge) to prevent access to the resource. In this case, the number of lunges beyond an initial response may then be expected to correlate with the level of frustration of the dog. Hence, for this measure in Test 2 (inability to access items), we could only use a subset of subjects, unlike the other behaviour measures in the remaining tests.

Other variations between these tests, worthy of discussion concern the influence of a handler on the measures of interest. In Test 2a, the dog was tethered on lead to a wall tie out, and a range of toys were out of reach. Frequency of lunging significantly correlated with barrier frustration (PC2) scores; however, vocalising did not. By contrast, in Test 5, the dog was on a lead and held by the experimenter, and withheld from accessing food thrown out of reach. In this test, frequency of vocalising significantly correlated with Frustration coping (PC5) scores; however, lunging did not. This might reflect an interspecific communicative function of the vocalisation, and the potential expectation of assistance from a handler when present in such contexts [39], with lunging serving as autonomous efforts to resolve the problem. This would offer a parsimonious explanation for both the significant and non-significant associations found in these tests and the current interpretation of the related PCs.

The lack of convergence between some behavioural measures and their proposed CFQ PC does not necessarily reflect a problem with the CFQ. Single behaviour test measures represent a snapshot of behaviour at a given time, and, accordingly, their predictive value has been questioned [40,41,42,43], i.e., the problem of predicting a trait from a single incident. By contrast, cumulative behavioural measures across the whole battery of tests (such as those used for vocalisation in this study) provide a result from a range of varied contexts related to the construct of interest, even if they are relatively closely aligned temporally. Such measures should therefore be considered better proxies of frustration tendencies: i.e., consistency in behavioural reactions across contexts [22,40,44].

Given the psychobiological basis to the interpretation of each PC, it was predicted that there would be a degree of discriminant validity in the selected behavioural measures across PCs 2–5 (see Supplementary Material Table S11). This was demonstrated for lunging in Test 2 (inability to access items) and barrier frustration (PC2) scores, and, scratching at the safety/baby gate in Tests 6a/6b (dog left alone) and autonomous control (PC4) scores, i.e., there was an absence of significant correlations with other PCs. However, vocalisation was less test specific and discriminative for both unmet expectation (PC3) and frustration coping (PC5) scores. However, for the latter (PC5), physiological changes (i.e., salivary cortisol change) relating to arousal during the behaviour test battery appear to be most discriminative. The lack of an association between salivary cortisol change and absolute post-test cortisol levels and the OQS suggests that this overall score from the CFQ provides much more than a simple measure of arousal.

While cortisol increase does not allow categorisation of valence of emotion experienced [45], there was convergence between various measures of frustration from the CFQ and behavioural tests and this physiological measure of arousal. This was demonstrated through the following: (1) salivary cortisol change occurred during a carefully designed frustration test battery, and correlated with frustration coping (PC5) scores; (2) dogs with absolute post-test cortisol >4 ng/mL had significantly higher frustration coping (PC5) scores (i.e., less able to cope with frustration); (3) dogs with absolute post-test cortisol >4 ng/mL vocalised in significantly more behaviour tests within the battery.

When assessing the pre-test cortisol levels, 17/39 dogs exceeded the arousal/”stress” threshold of 4 ng/mL threshold [30,31]. There are several potential reasons for this: factors related to the study environment could have played a role in high pre-test cortisol, e.g., fear of the experimenter, owner separation, and being in a novel environment could all be implicated in increasing arousal [46]. However, all dogs were sufficiently motivated to work for food during the study, and were amenable to saliva sampling without restraint, which suggests that significant fear of the experimenter was not the primary cause. Whilst it is possible that some dogs have persistent elevated salivary cortisol levels, it is also likely that insufficient time to relax after travelling, or to habituate to the test room may have contributed to these findings. To overcome this, some studies have assessed baseline cortisol levels at a different time and environment where the dog is calm [30,47]. A limitation of this approach is the potential for a ‘ceiling effect’—i.e., on the day of testing, a higher than expected baseline cortisol level immediately before testing could alter the extent to which salivary cortisol levels may change as well as absolute post-test cortisol levels, and the researcher would be unaware [48,49,50]. This was the rationale for measuring salivary cortisol levels immediately before testing, and excluding the physiological data from those dogs with elevated pre-test cortisol. If this study were to be repeated in the future, it is suggested that the protocol be modified to allow dogs a period of rest following travel, and lengthen the period of habituation prior to the test battery, in an attempt to minimise the proportion of dogs with high pre-test cortisol levels. Alternatively, owners could be recruited to attend a range of habituation visits prior to testing, as undertaken by Beerda, et al. [51], where dogs were habituated to the test room over two days. Whilst this may reduce the overall novelty of the test room, including interactions with the experimenter, such additional time commitment required from owners may reduce the likelihood of recruiting sufficient subjects for analysis.

## 5. Conclusions

Convergent and discriminant validity was demonstrated with a range of behavioural test measures and owner report from the CFQ OQS and PC2-5 scores. As expected, associations were demonstrated between CFQ measures and salivary cortisol change during the test battery, providing evidence of convergent validity between the CFQ and this physiological measure of arousal. Overall, the demonstration of convergent validity with owner independent behavioural and physiological measures increases confidence in using the CFQ for the objective quantification of frustration tendencies through owner report.

## Figures and Tables

**Table 1:** Details and durations of frustration tests within test battery. For those based on existing tests, source, and modification(s) are stated.

Test No.	Test Name	Summary of Details of Test	Source/Modification in Proposed Test	Duration (Minutes)
1a	High value (4 treats)	Dog’s name called, offered 4 small treats. Repeated 5 times in total, 10 s interval between trials—delayed reward	[7,20] Downshift in quantity rather than quality; reduced number of trials/test duration	2
1b	Low value (1 treat)	Dog’s name called, then offered 1 small treat. Repeated 5 times in total, 10 s interval between trials—reduced reward		
1c	No treat	Dog’s name called, then offered empty hand/no treat. Repeated 3 times in total, 10 s interval between trials—absent reward		
2a	Inability to access items	Dog attached to wall tie out by lead. A range of toys are placed out of reach by experimenter.	Developed de novo	2
2b	Ease of removal of a range of items	After accessing items for 30 s, experimenter removes items.		
3	Delay in leaving a room when lead clipped on	Lead clipped on dog, safety/baby gate partially opened and motion to leave room, however experimenter remains stationary for 1 min.	Developed de novo	1
4	Dog ignored whilst in test room	Experimenter sits on sofa in room reading book for 5 min ignoring dog.	Developed de novo	5
5	Ability to access food denied, restrained by lead	Dog on lead held by experimenter. Treat thrown in front of dog, dog permitted to access it. Repeated 5 times in total, 10 s interval between trials. 6th treat thrown beyond reach of dog, not permitted access to it for 1 min.	[29] Increased number of treats before access denied; thwarted by lead rather than body blocking	2
6a	Left alone, experimenter out of sight	Experimenter leaves dog in test room for 30 s, out of sight of dog, outside test room.	[29] Left alone by experimenter rather than owner, in room rather than tethered	1
6b	Left alone, experimenter in sight	Experimenter leaves dog in test room for 30 s, in sight of dog separated by safety/baby gate.		

**Table 2:** Ethogram detailing behavioural measures and corresponding tests selected for correlational analysis with CFQ.

Category	Behaviour	Definition	Measure	Test
Vocalising	Bark	Staccato vocalization	Frequency	ALL
	Whine	Prolonged moan	Frequency	ALL
Barrier related behaviour	Lunge (on lead)	When on lead a forward movement whereby the dog reaches the full extent of the lead	Frequency	Tests 2 and 5
	Paw/scratch gate	Pawing/scratching movement of a front paw on the safety/baby gate	Frequency	Test 6a/6b
Ambulation	Ambulating	Action of walking or running for at least 2 steps	Duration	Test 4
Body posture (when stationary)	Sit	Sitting with hind legs in a flexed position and front legs in a stretched position (may be rest against a wall).	Duration	
	Lying down	Lying in a lateral or a ventral position, with head up or down and eyes open or closed.	Duration	
	Stand	Standing position, supported by 3 or 4 legs.	Duration	

**Table 3:** Comparison of CFQ/PC scores and vocalising measures from behaviour test battery between ‘post-test cortisol <4.0 ng/mL group’ and ‘post-test cortisol >4.0 ng/mL group’ using Mann–Whitney tests.

Measure	Median Score		Mann Whitney *U*	*Z*	Exact Sig. (2 Tailed)
	Post-Test Cortisol < 4.0 ng/mL Group (*n* = 15)	Post-Test Cortisol > 4.0 ng/mL Group (*n* = 7)			
CFQ OQS	0.45	0.51	31.500	−1.482	0.138
CFQ PC1	0.44	0.40	52.500	0.000	1.000
CFQ PC2	0.55	0.70	32.000	−1.450	0.147
CFQ PC3	0.55	0.65	35.000	−1.241	0.214
CFQ PC4	0.32	0.44	31.500	−1.491	0.136
CFQ PC5	0.40	0.53	21.500	−2.219	0.027 *
Vocalising—total number of tests	3.0	7.0	17.500	−2.156	0.031 *
Vocalising—total frequency (all tests)	10.0	110.5	26.000	−1.481	0.139

* Significant at *p* < 0.05 level. CFQ = Canine frustration questionnaire; OQS = Overall questionnaire score; PC = principal component, where: PC1 (‘General frustration’); PC2 (‘Barrier frustration/perseverance’); PC3 (‘Unmet expectations’); PC4 (‘Autonomous control’); PC5 (‘Frustration coping’).

## Data Availability

The data presented in this study are openly available in https://osf.io/s2mb8/?view_only=0ebb6efb240147b2b882b3365623c1af (accessed on: 10 November 2021).

## Supplementary Materials

The following are available online at https://www.mdpi.com/article/10.3390/ani11123346/s1, Figure S1: Behaviour Test Protocol; Table S1: Proposed mapping of behaviour test measures onto CFQ items, for OQS/PC1; Table S2: Proposed mapping of behaviour test measures onto CFQ items, for PC2; Table S3: Proposed mapping of behaviour test measures onto CFQ items for PC3; Table S4: Proposed mapping of behaviour test measures onto CFQ items for PC4; Table S5: Proposed mapping of behaviour test measures onto CFQ items for PC5; Table S6: Counterbalanced test order schedule for Group 1—‘odd’ group; Table S7: Counterbalanced test order schedule for Group 2—‘even’ group; Table S8: Intraclass correlation coefficient (ICC) for inter-rater reliability assessment of behavioural measures coded from test footage; Table S9: Comparison of CFQ OQS/PCs between counterbalanced groups using Mann Whitney tests; Table S10: Comparison of key behavioural measures between counterbalanced groups using Mann Whitney tests; Table S11: Spearman’s rank order correlations between Canine Frustration Questionnaire (CFQ) principal components (PC) 2 to 5, and paired behavioural measure demonstrating convergent validity; Table S12: Comparison of CFQ OQS/PCs between ‘behaviour test group’ and ‘questionnaire generation group’ using Mann Whitney tests; Table S13: Spearman’s rank order correlations between CFQ OQS/PCs, vocalising measures from behaviour test battery and cortisol levels (pre-test, post-test and pre-test to post-test change. Video S1: Behaviour test protocol footage.
