# Supplementary material for: Behavioural and Physiological Correlates of the Canine Frustration Questionnaire
Source: Animals (Basel). 2021 Nov 23;11(12):3346. doi: 10.3390/ani11123346 (PMC8698056; doi:10.3390/ani11123346)
Supplement: Supplementary file 1 [file animals-11-03346-s001.zip › animals-1425262- Supplementary part 1 - figure -for proofreading KM.pdf]

animals

MDPI

Supplementary Materials

Behavioural and Physiological Correlates of the Canine Frustration Questionnaire

Kevin J. McPeake [1,2,*], Lisa M. Collins [3], Helen Zulch [2] and Daniel S. Mills [2]

Citation: McPeake, K.J.; Collins, L.M.; Zulch, H.; Mills, D.S. Behavioural and Physiological Correlates of the Canine Frustration Questionnaire. *Animals* **2021**, *11*, 3346. https://doi.org/10.3390/ani11123346

Academic Editor: Björn Forkman

Received: 30 September 2021
Accepted: 18 November 2021
Published: 23 November 2021

Publisher's Note: MDPI stays neutral with regard to jurisdictional claims in published maps and institutional affiliations.

Copyright: © 2021 by the authors. Submitted for possible open access publication under the terms and con-

ditions of the Creative Commons Attribution (CC BY) license (https://creativecommons.org/licenses/by/4.0/).

[1] The Royal (Dick) School of Veterinary Studies, Easter Bush Campus, Midlothian, EH25 9RG, UK
[2] Animal Behaviour Cognition and Welfare Group, School of Life Sciences, University of Lincoln, Lincoln, LN6 7TS, UK; hzulch@lincoln.ac.uk (H.Z.); dmills@lincoln.ac.uk (D.S.M.)
[3] Faculty of Biological Sciences, University of Leeds, Leeds, LS2 9JT, UK; l.collins@leeds.ac.uk
* Correspondence: kevin.mcpeake@ed.ac.uk

Figure S1 - Behaviour Test Protocol

- Experimenter will meet the dog and owner outdoors
- Owner brings the dog into the test room with experimenter, and allowed off lead to explore and habituate to the test room and experimenter (with owner present)
- Owner signs the consent form allowing their dog to take part in the study
- Dogs must wear a non-tightening flat collar or harness to participate, and if an owner does not use one, these will be provided and fitted prior to commencing the behaviour tests
- Owner asked to leave the room via the door entered by
- Owner asked to complete short questionnaire and Canine Frustration Questionnaire whilst in another room
- Where consent provided, pre-test saliva sample taken from dog

Duration of habituation to experimenter and room ~10 minutes

Potential reasons for exclusion / or limited inclusion

- If the dog does not permit the collection of a saliva sample, (failure to approach experimenter, body language indicating concerning levels of fear/anxiety/frustration etc) then they may proceed with the behavioural tests without such physiological measures being taken
- If the dog fails to habituate to the test room they will be excluded from the study
- If the dog does not eat, or is not motivated to engage with the experimenter whilst separated from the owner, then they will be excluded on a test by test basis

Test 1. Downshift high value to low value to no treat

- In test room, dog on 2 metre lead attached to wall tie out
- Experimenter sits 1 metre to side of dog, so dog can access experimenter on loose lead, but can also choose to move up to 3 metres from experimenter if so desired

<u>Test 1a</u>

- Dog's name called, then offered <u>4 small treats</u> from experimenter's hand – the food remains on offer for 5 seconds. If not consumed, the food is returned to the pot
- This is repeated 4 times (so, 5 times in total), with an interval of 10 seconds between food being consumed/returned to pot and dog's name being called

<u>Test 1b</u>

- Dog's name called, then offered <u>one small treat</u> from experimenter's hand – the food remains on offer for 5 seconds. If not consumed, the food is returned to the pot
- This is repeated 4 times (5 times in total), with an interval of 10 seconds between food being consumed/returned to pot and dog's name being called

<u>Test 1c</u>

- Dog's name called, then <u>no food treat given</u> – experimenter shows empty hand, hand remains open for 5 seconds before closing hand and moving it away from dog
- This is repeated 2 times, with an interval of 10 seconds between calling the dog and empty hand being offered

Dog's name called, then offered <u>one small treat</u> from experimenter's hand – the food remains on offer for 5 seconds. If not consumed, the food is returned to the pot. The purpose of this final treat both to offset any frustration experienced by non-reward in previous two trials and serves as a control to ensure that satiety has not been reached.

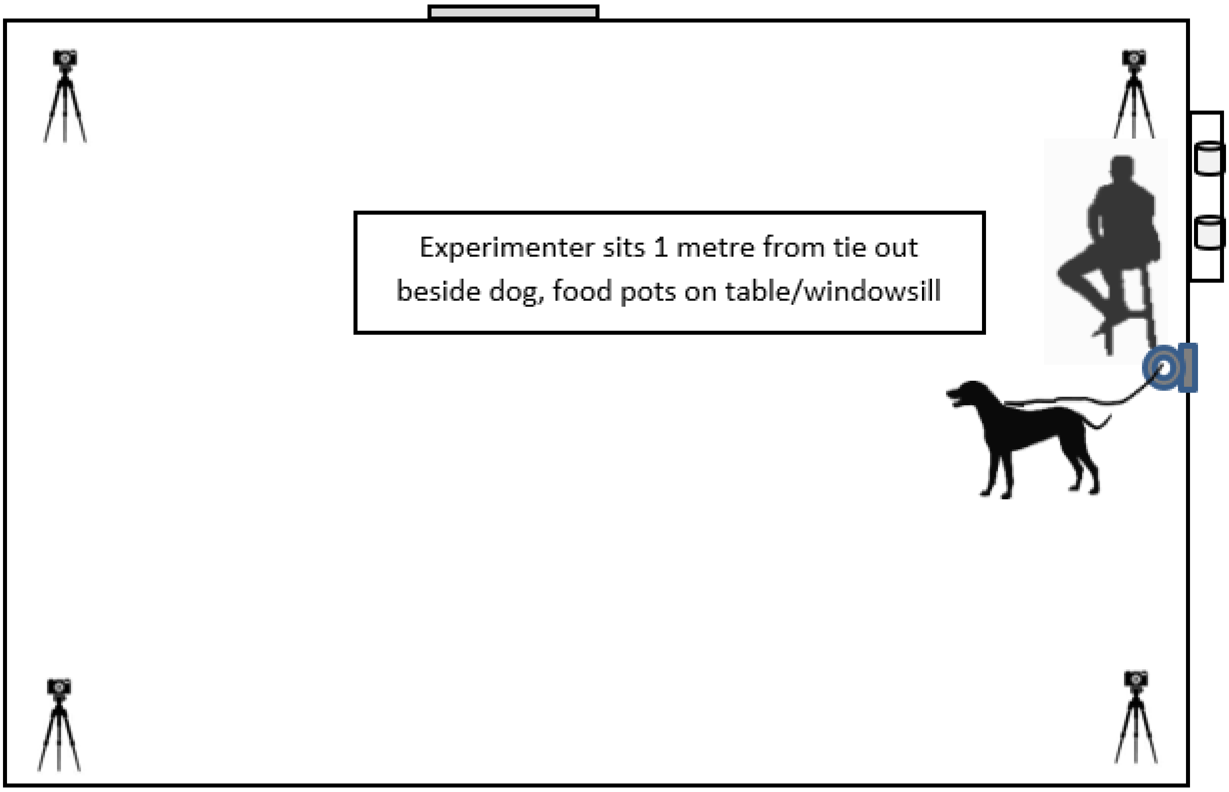

Break – 2 minutes, on lead, experimenter holds lead, allows dog to explore/lie down etc

Test 2a. Inability to access items

- In test room, dog on lead attached to wall tie out
- Experimenter collects range of toys from box on table– squeaky tennis ball, tug toy, frisbee, rubber Kong with piece of food dog can tolerate in (single piece of food, easy to remove if Kong dropped will fall out)

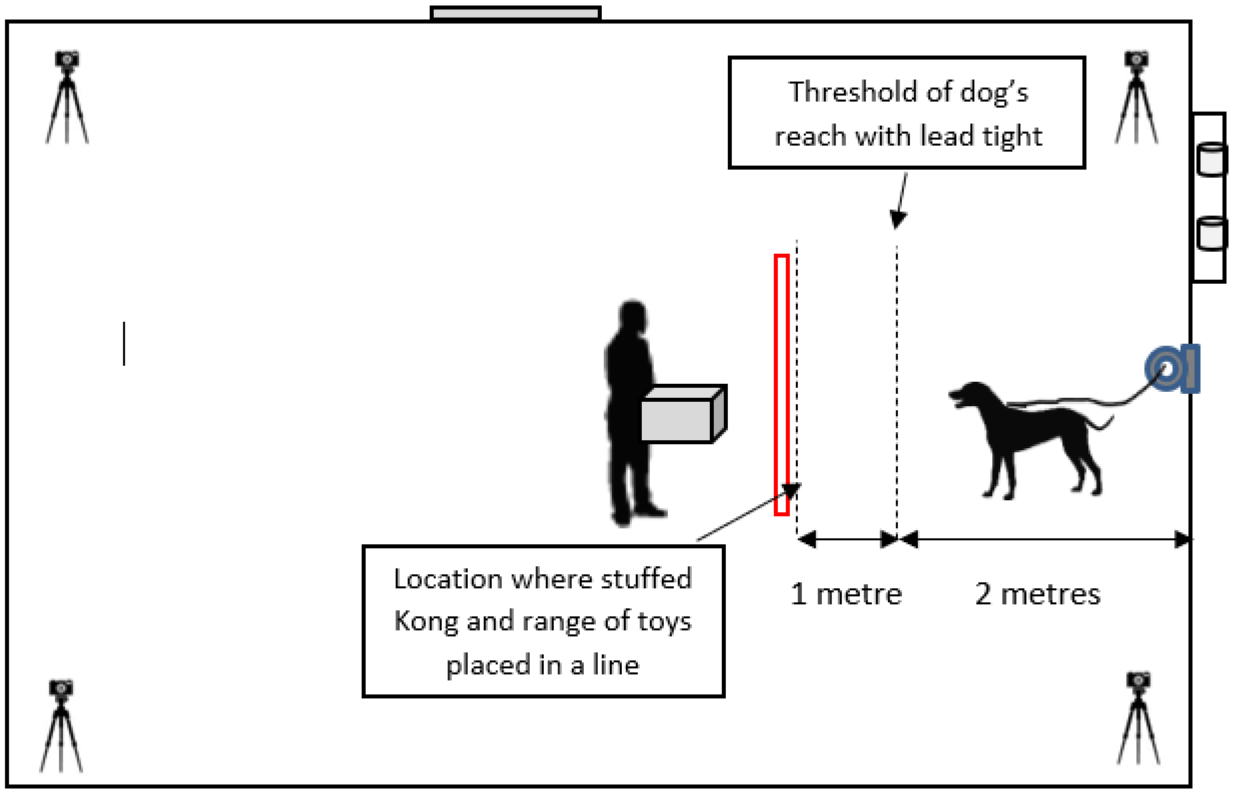

- Experimenter makes eye contact with the dog and says dog's name once as each toy is put down to increase interest in the toys, including squeaking ball once as placed on ground. Items placed on floor in sight of dog 3 metres from tie out (1 metre from dog on 2 metre lead)
- Experimenter moves to right side of dog, 1 metre from wall tie-out

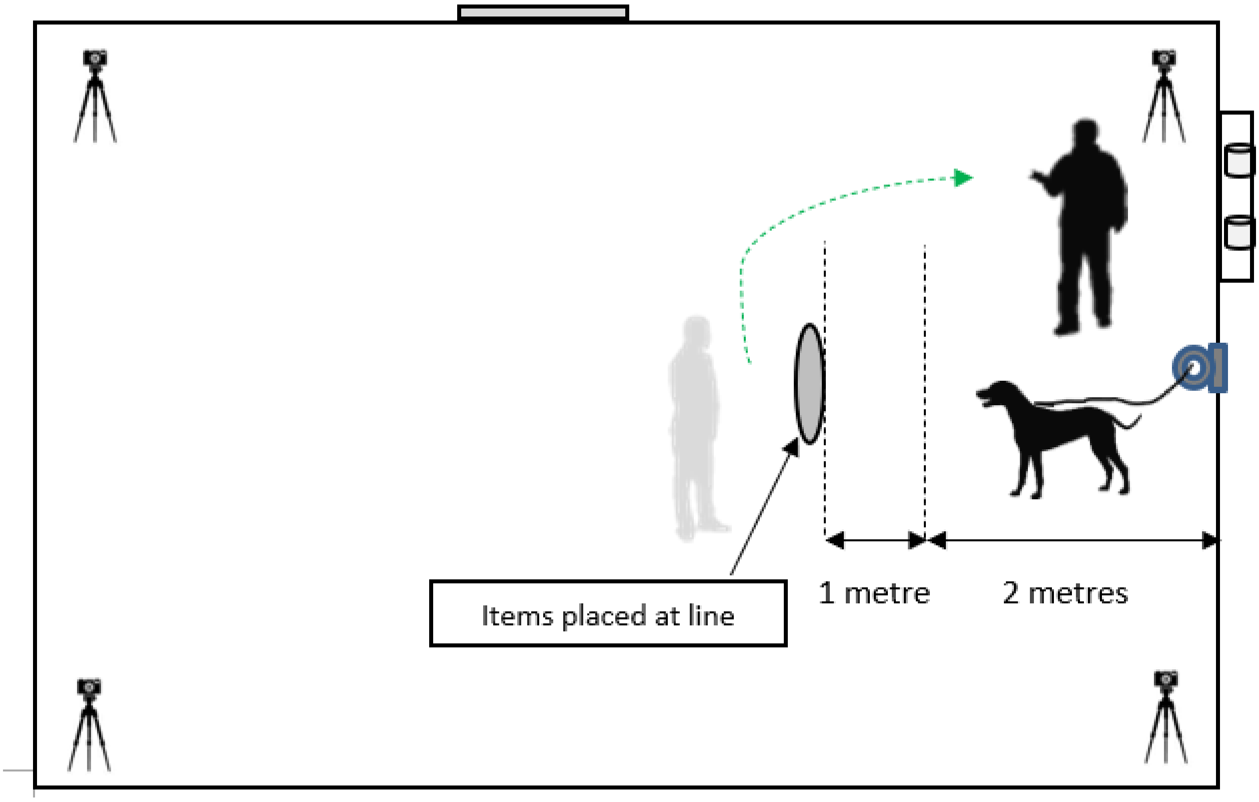

- After 10 seconds, experimenter calls dog's name once
    - If dog comes to experimenter, food treat given, dog lead unclipped, allowed to go to toys etc
    - If dog does not come to experimenter, wait 10 seconds, experimenter calls dog's name a second time – if dog comes to experimenter, food treat given, dog lead unclipped, allowed to go to items
    - If dog does not come to experimenter, wait 10 seconds, experimenter calls dog's name a third time – if dog comes to experimenter, food treat given, dog lead unclipped, allowed to go to items
    - If dog does not come to experimenter, experimenter moves towards dog, shows food treat, lures dog away from items towards where experimenter was, dog given food treat, lead unclipped from dog and dog allowed to go to items
- Dog permitted to access and interact with items for 30 seconds

Test 2b. Ease of removal of a range of items

- In test room, dog off lead

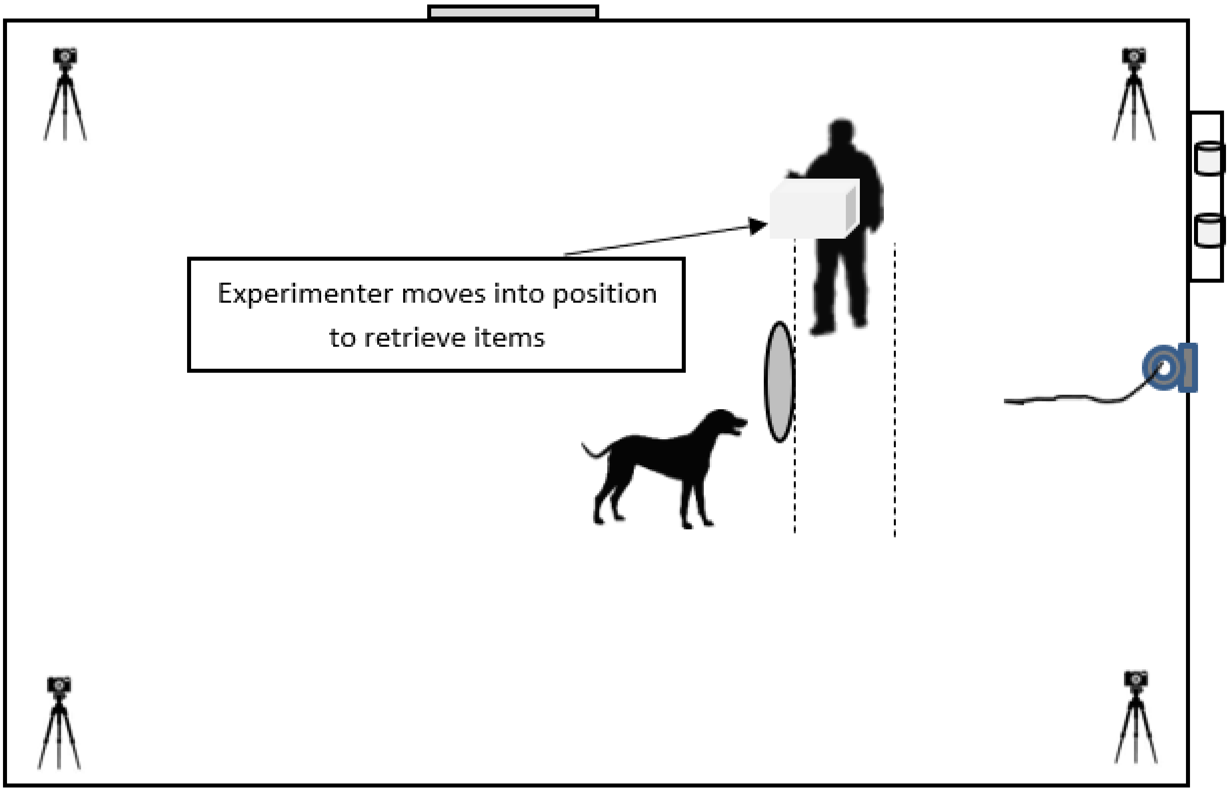

- At end of 30 seconds of being permitted to interact with items:
    - If dog no longer interested in items, experimenter retrieves all items on floor to put back into box from further from dog to closest to dog, then picks up dog's lead, moves towards dog clips lead on, treat given, proceed to break
    - If dog still interested in items/engaging with them, experimenter moves towards items:
        - If dog is oriented near items, but does not have one in mouth, experimenter retrieves all items on floor to put back into box from further from dog to closest to dog
            - If dog has item in mouth the experimenter retrieves all items on floor to put back into box from further from dog to closest to dog, then experimenter stretches out hand to retrieve item from mouth.
                - If dog drops/relinquishes toy, experimenter removes it, puts it back in box, clips on dog lead, gives food treat, proceed to break
            - If dog does not relinquish item from mouth, experimenter produces food treat from pocket to try and swap for item – if drops, food given, item removed and put back in box
                - Experimenter returns to room, clips on dog lead, gives food treat, proceed to break
            - If dog still does not relinquish item, experimenter moves away from dog, to second box of identical toys (including Kong with food) out of reach of dog. Experimenter produces equivalent item, shows interest in it, attempt to get dog to move into

corridor through door, by luring with item, food tossed for dog, experimenter retrieves item from test room whilst dog secured in corridor

        - Experimenter returns to room, clips on dog's lead, gives food treat, proceed to break

    - If dog still does not relinquish the toy, toy left with dog for longer period until loses interest – if this does not happen, then leave toy with the dog

- Test situation stopped if dog shows aggressive/threatening behaviours (growling, snarling, snapping, biting, lunging) to experimenter, in which case experimenter moves away from dog, to second box of identical toys (including Kong with food) out of reach of dog. Experimenter produces equivalent item, shows interest in it, attempt to get dog to move into corridor through door, by luring with item, food tossed for dog, experimenter retrieves item from test room whilst dog secured in corridor
- Ability to move into other room (i.e. corridor) if concerning aggressive behaviours

Break – 2 minutes, on lead, experimenter holds lead, allows dog to explore/lie down etc

Test 3. Delay in leaving a room when lead clipped on

- In test room, dog on lead held by experimenter
- Prepare dog to leave test room by walking to corridor/test room door opening, experimenter puts hand on safety/baby gate, and opens approximately 3-5cm as if to leave, but does not open it
- Wait 1 minute
- Behaviour monitored for 1 minute whilst in this position

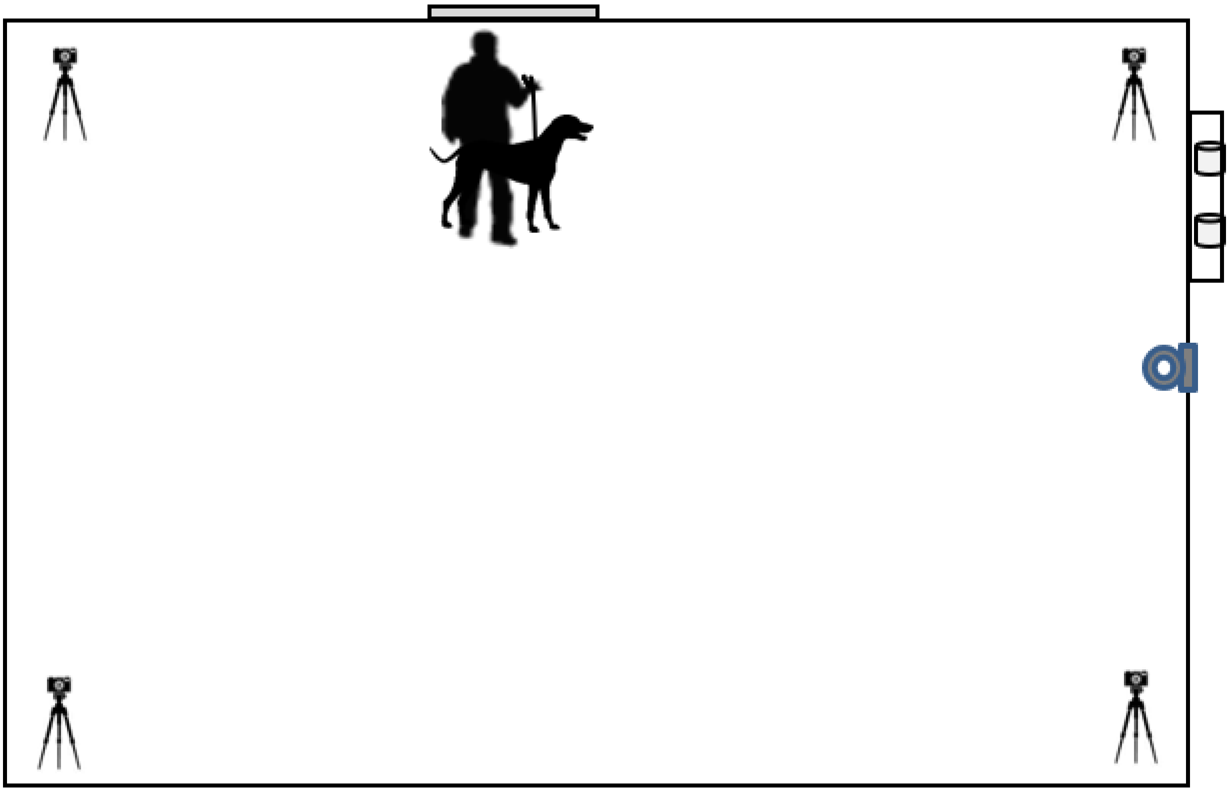

- At end of 1 minute
  - if dog calm/not jumping/scratching/vocalising, safety/baby gate opened, experimenter goes through door with dog
  - If dog jumping and/or showing other undesirable behaviours, experimenter will try and distract, speak to, request alternative behaviour, and when showing more appropriate behaviour, safety/baby gate open and both go into corridor
- Test situation stopped If dog shows aggressive/threatening behaviours (growling, snarling, snapping, biting, lunging) to experimenter, in which case experimenter will try and request alternative behaviours before rewarding with food from pocket, and/or allow access to food/drop lead depending on safety
- Ability for experimenter to move into other room (i.e. corridor) if concerning aggressive behaviours

Additional measures? - Record observable signs related to frustration towards barrier, as well as towards experimenter and lead, and need for requesting an alternative behaviour at end of 1 minute.

Break – ~5 minutes - Dog taken outdoors on lead, opportunity to wander around, toilet

Test 4. Dog ignored whilst in test room

- Return to test room, experimenter and dog
- Dog filmed for 5 minutes whilst off lead and nothing provided, experimenter does not interact (experimenter reads a book, sitting on chair – note, experimenter to sit on a sofa – this is a different chair/different position to that where dog expected to receive food treats in test 1, to minimise any confounding factors for dogs in group 1, where test 1 comes before the break)

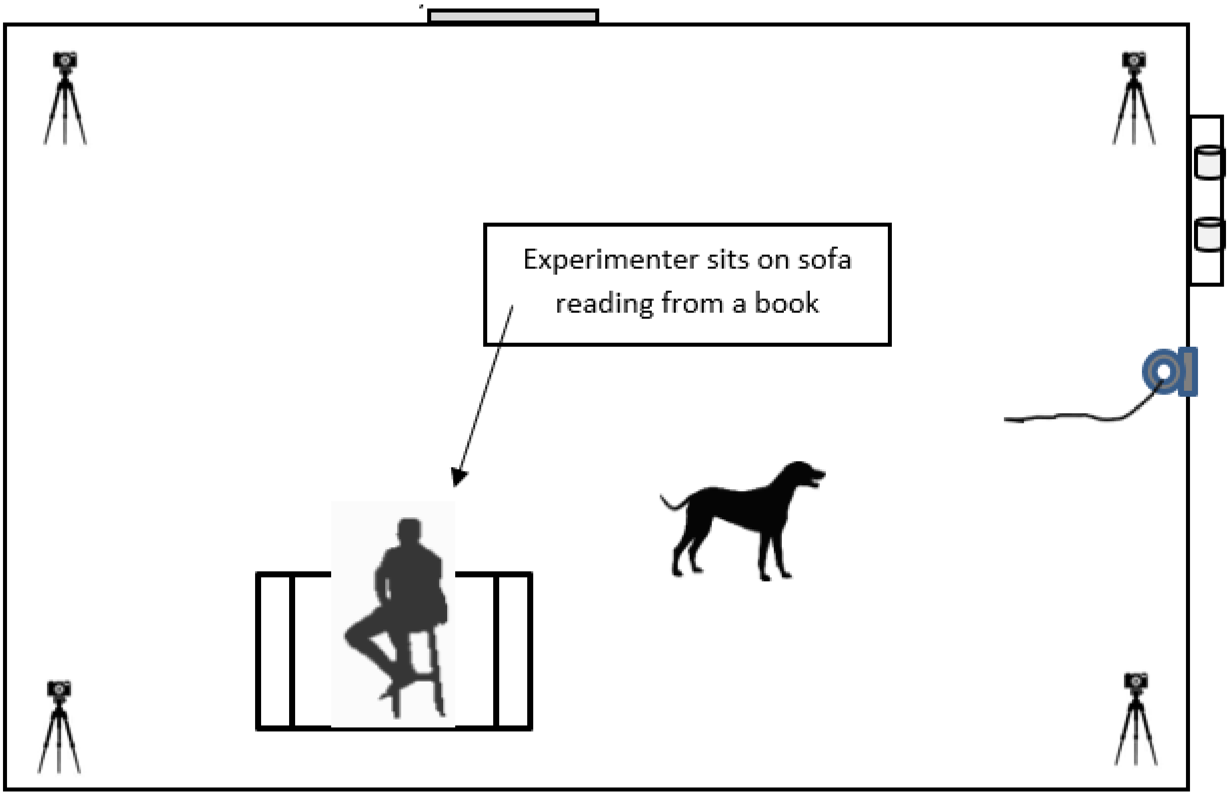

Test 5. Ability to access food denied, restrained by lead

- In test room, dog on lead held by experimenter

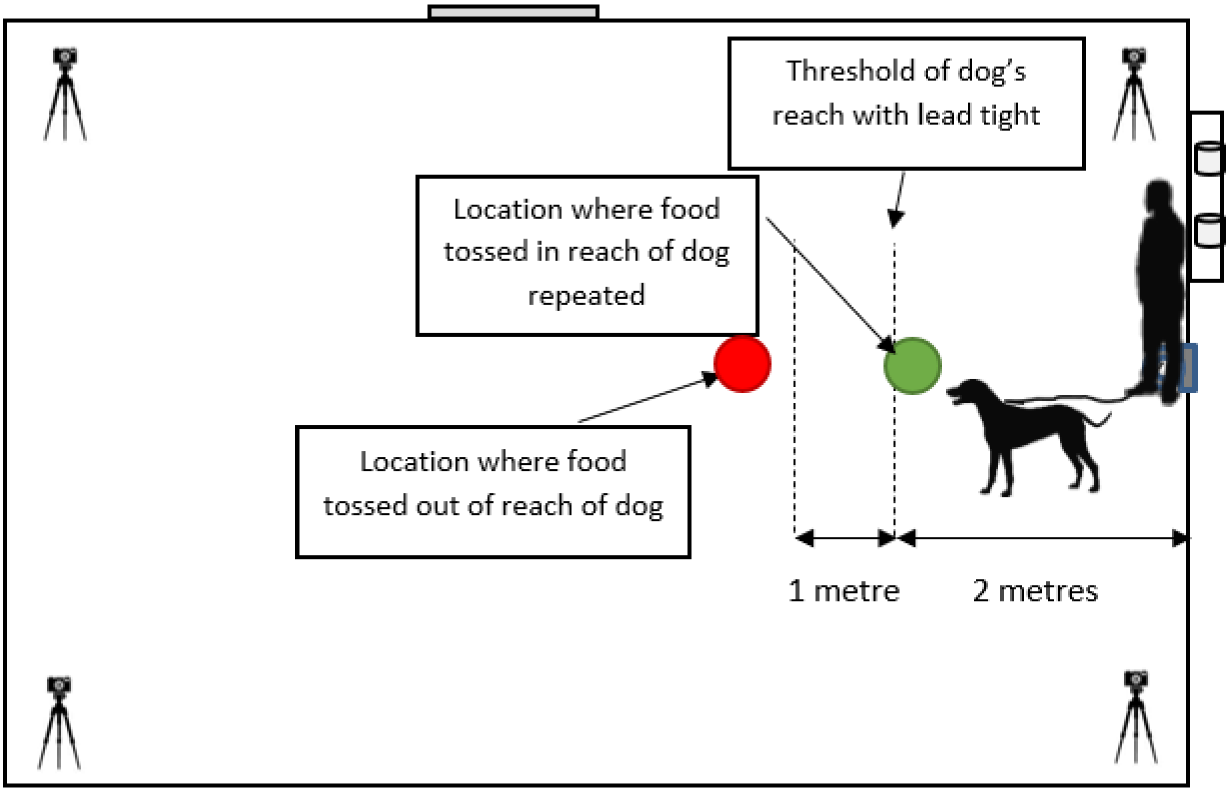

- Piece of food tossed by experimenter in front of dog, dog allowed to eat food. Repeated 5 times in quick succession approximately every 10 seconds (different dogs may take different amounts of time to eat) – food is large enough dog can see clearly, and sticky and not round, so does not bounce or roll away
- Piece of food tossed in front of dog just out of reach (around 1 metre from where dog can reach at extent of lead), dog not allowed to get to it.
- Behaviour recorded/monitored for 1 minute whilst not allowed to get food.
- At end of 1 minute
    - if dog calm/not pulling – can progress to picking up food
    - If dog pulling and or showing other undesirable behaviours, experimenter will try and distract, speak to, request alternative behaviour, reward this, then when not showing undesirable behaviour will quickly allow the dog to retrieve original out of reach food
- Test situation stopped If dog shows aggressive/threatening behaviours to experimenter, in which case experimenter will try and request alternative behaviours before rewarding with food from pocket, and/or allow access to food/drop lead depending on safety
- Ability to move into other room (i.e. corridor) if concerning aggressive behaviours

Break – 2 minutes, off lead, dog allowed to explore the room

Test 6a. Left alone in a room, experimenter out of sight

- In test room, dog off lead
- Single food treat tossed for dog to retrieve, so moves away from door as experimenter leaves room and secures safety/baby gate so dog cannot follow
- Experimenter leaves small corridor to test room, then exits door to main corridor and moves out of sight of dog in the main corridor for 30 seconds

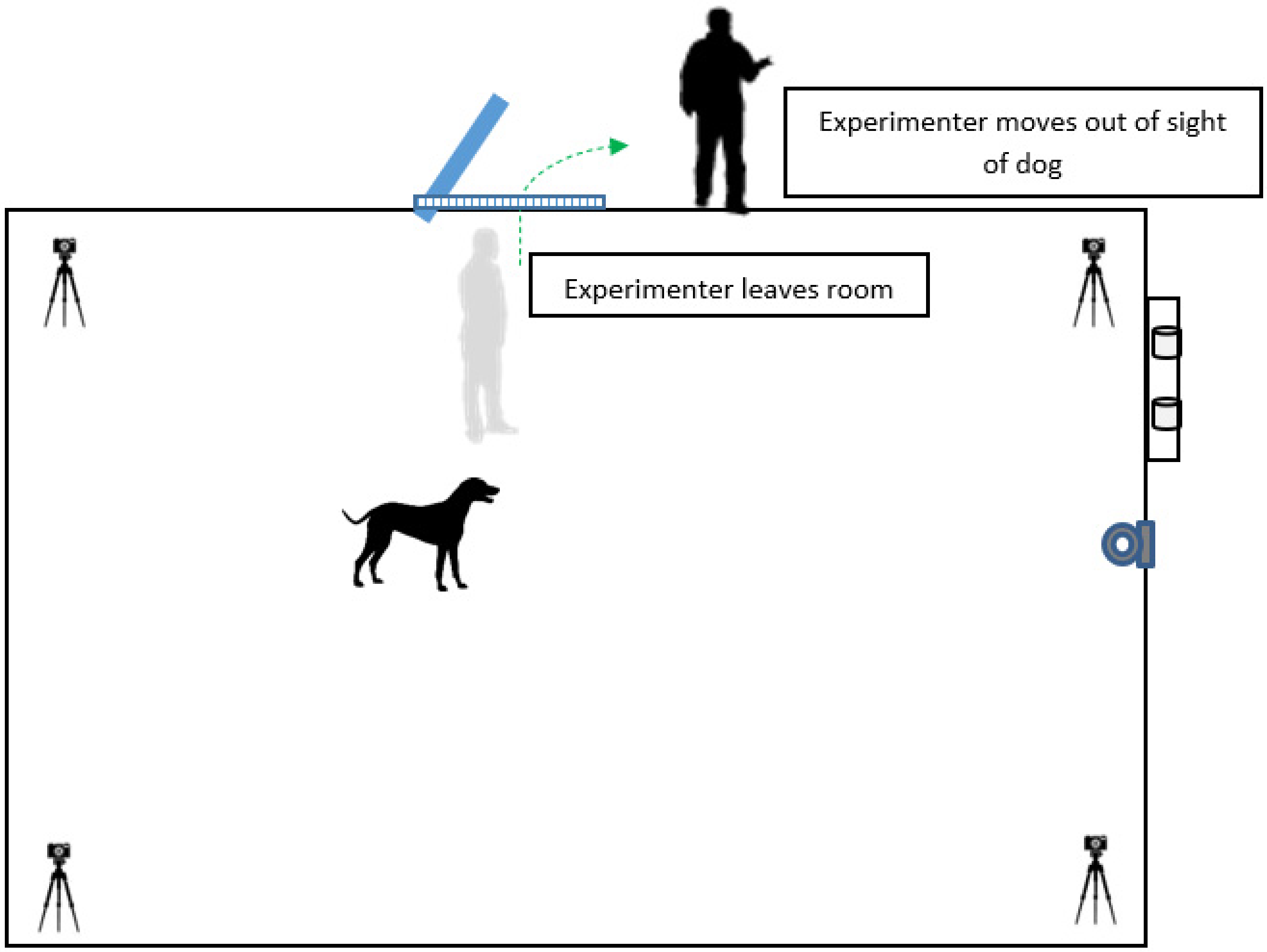

Test 6b. Left alone in a room, experimenter in sight

- Experimenter leaves small corridor to test room, then exits door to main corridor and moves out of sight of dog in the main corridor for 30 seconds
- Experimenter re-enters door from main corridor, and remains in small corridor 1 metre from safety/baby gate so in sight of dog

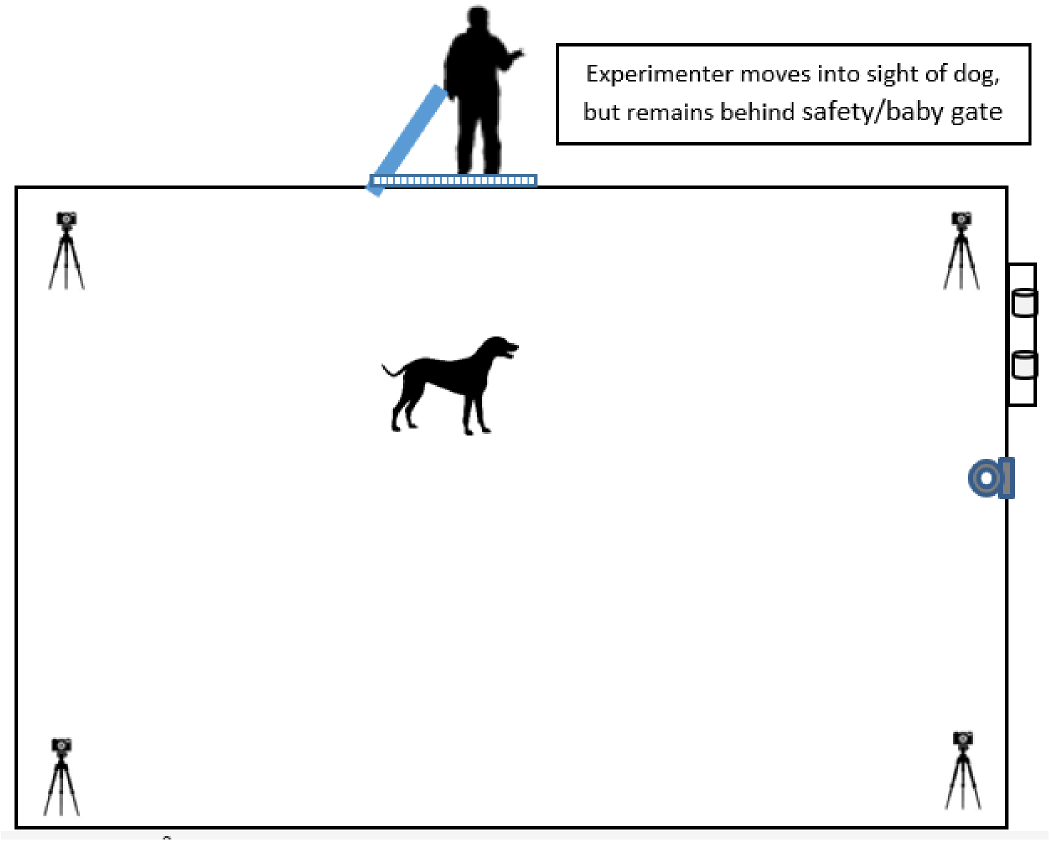

- Experimenter remains in this position for 30 seconds looking towards dog and will continue to look through the safety/baby gate for duration
- At end of full 1 minute of separation:
    - If dog calm/relaxed whether at gate or not, experimenter opens gate, re-enters room, no fuss given to dog
    - If dog displaying undesirable behaviours (e.g. vocalising, barking, scratching door) experimenter will approach door and wait 5 seconds for more desirable behaviour
        - If more desirable behaviour displayed (e.g. sit/stand/down, quiet, scratching ceases) experimenter opens door, re-enters room, no fuss given to dog
    - If dog continues to display undesirable behaviour experimenter will request a known behaviour e.g. 'sit' and if sits within 5 seconds experimenter opens door, re-enters room, no fuss given to dog
    - If dog continues to display undesirable behaviour, door opened regardless of behaviour, experimenter re-enters room, no fuss given to dog

END OF TEST

2 minutes then 2nd saliva swab, dog returned to owner
