# Supplementary material for: Behavioural and Physiological Correlates of the Canine Frustration Questionnaire
Source: Animals (Basel). 2021 Nov 23;11(12):3346. doi: 10.3390/ani11123346 (PMC8698056; doi:10.3390/ani11123346)
Supplement: Supplementary file 1 [file animals-11-03346-s001.zip › animals-1425262- Supplementary part 2 - Tables-for proofreading KM.pdf]

animals

Supplementary Materials

Behavioural and Physiological Correlates of the Canine Frustration Questionnaire

Kevin J. McPeake [1,2,*], Lisa M. Collins [3], Helen Zulch [2] and Daniel S. Mills [2]

Citation: McPeake, K.J.; Collins, L.M.; Zulch, H.; Mills, D.S. Behavioural and Physiological Correlates of the Canine Frustration Questionnaire. *Animals* **2021**, *11*, 3346. https://doi.org/10.3390/ani11123346

Academic Editor: Björn Forkman

Received: 30 September 2021
Accepted: 18 November 2021
Published: 23 November 2021

Publisher's Note: MDPI stays neutral with regard to jurisdictional claims in published maps and institutional affiliations.

Copyright: © 2021 by the authors. Submitted for possible open access publication under the terms and con-

ditions of the Creative Commons Attribution (CC BY) license (https://creativecommons.org/licenses/by/4.0/).

[1] The Royal (Dick) School of Veterinary Studies, Easter Bush Campus, Midlothian, EH25 9RG, UK
[2] Animal Behaviour Cognition and Welfare Group, School of Life Sciences, University of Lincoln, Lincoln, LN6 7TS, UK; hzulch@lincoln.ac.uk (H.Z.); dmills@lincoln.ac.uk (D.S.M.)
[3] Faculty of Biological Sciences, University of Leeds, Leeds, LS2 9JT, UK; l.collins@leeds.ac.uk
* Correspondence: kevin.mcpeake@ed.ac.uk

Table S1. Proposed mapping of behaviour test measures onto CFQ items, for OQS/PC1.

	**Item Mapping Onto Behaviour Tests**							
Principal component	Item	Test 1 Downshift 4 treats (1a), 1 treat (1b), 0 treats (1c)	Test 2 Inability to access items (2a) Ease of removal of a range of items (2b)	Test 3 Delay in leavin g a room	Test 4 Dog ignored whilst in test room	Test 5 Ability to access food denied, restrained by lead	Test 6 Left alone in a room, experimenter out of sight (6a); in sight (6b)	Selected behaviour test measure(s) to validate principal component(s)
OQS	ALL	✔	✔	✔	✔	✔	✔	<u>OQS and PC1 scores</u><br>and<br><u>All test sections</u> (1a,1b,1c,2a,3,4,5,6a,6b)<br>-Number of tests where vocalising (barking/whining) occurs<br>-Total frequency of vocalising (barking/whining) across all tests
PC1: General frustration	My dog becomes frustrated in a large range of situations	✔	✔	✔	✔	✔	✔	
	There are days when my dog seems to become more easily frustrated than others for no apparent reason							
	My dog appears to become frustrated frequently (e.g. at least once daily)							
	My dog shows increases in certain behaviours (e.g. lip licking, yawning, mounting, full body shake off) if he/she cannot immediately access something they want	✓	✓	✓		✓		
	My dog engages in a repetitive behaviour (e.g. tail chasing, pacing, circling) when unable to access something he/she wants	✓	✓	✓	✓	✓	✓	

(✔ = suggested strong mapping of item; ✓ = potential mapping of item).

Table S2. Proposed mapping of behaviour test measures onto CFQ items, for PC2.

Principal component	Item	Item mapping onto behaviour tests: Test 1 Downshift 4 treats (1a), 1 treat (1b), 0 treats (1c)	Test 2 Inability to access items (2a) Ease of removal of a range of items (2b)	Test 3 Delay in leaving a room	Test 4 Dog ignored whilst in test room	Test 5 Ability to access food denied, restrained by lead	Test 6 Left alone in a room, experimenter out of sight (6a); in sight (6b)	Selected behaviour test measure(s) to validate principal component(s)
PC2: Barrier frustration / perseverance	My dog shows continued efforts (e.g. lunging, pulling towards) to approach a dog/person they wish to greet, when being restrained from doing so (e.g. when on lead)						✓	Test 2a<br>-Lunging frequency<br>-Vocalising (barking/whining) frequency
	When on lead my dog will persist in lunging/pulling towards something he/she would like to chase (e.g. a cat, rabbit, bird, toy)		✔			✓		
	My dog has difficulty in responding to cues/commands (e.g. sit, lie down, stay) if there is something else he/she wants to do or access		✓					
	My dog gets upset if shut away from visitors (e.g. vocalises or scratches/digs at the door)						✓	

(✔ = suggested strong mapping of item; ✓ = potential mapping of item).

Table S3. Proposed mapping of behaviour test measures onto CFQ items for PC3.

	**Item mapping onto behaviour tests**							
Principal component	Item	Test 1 Downshift 4 treats (1a), 1 treat (1b), 0 treats (1c)	Test 2 Inability to access items (2a) Ease of removal of a range of items (2b)	Test 3 Delay in leaving a room	Test 4 Dog ignored whilst in test room	Test 5 Ability to access food denied, restrained by lead	Test 6 Left alone in a room, experimenter out of sight (6a); in sight (6b)	Selected behaviour test measure(s) to validate principal component(s)
PC3: Unmet expectations	My dog does not like being left out of activities with other dogs							<u>Test 3</u><br>-Vocalising (barking/whining) frequency<br><u>Test 4</u><br>-Duration of ambulatory behaviour
	My dog appears agitated and unsettled when he/she wants something another dog has (e.g. a toy or food item)							
	My dog becomes very excited/restless (e.g. pacing, whining, barking, jumping up) when waiting to take part in an enjoyable activity		✓	✔	✔	✓		
	My dog appears unsettled when there are delays in his/her routine (e.g. if walked or fed later than usual)			✓				

(✔ = suggested strong mapping of item; ✓ = potential mapping of item).

Table S4. Proposed mapping of behaviour test measures onto CFQ items for PC4.

Principal component	Item mapping onto behaviour tests							Selected behaviour test measure(s) to validate principal component(s)
	Item	Test 1 Downshift 4 treats (1a), 1 treat (1b), 0 treats (1c)	Test 2 Inability to access items (2a) Ease of removal of a range of items (2b)	Test 3 Delay in leaving a room	Test 4 Dog ignored whilst in test room	Test 5 Ability to access food denied, restrained by lead	Test 6 Left alone in a room, experimenter out of sight (6a); in sight (6b)	
PC4: Autonomous control	My dog becomes aggressive (i.e. growl, snap or bite) if I try to remove an item he/she has (e.g. favourite toy or food)		✔					<u>Test 2b</u> -Frequency of aggressive behaviours<br><br><u>Test 6a/6b</u> -Frequency of pawing/scratching at baby gate
	When my dog is not kept busy, he/she can repeatedly lick, chew or nibble their own body parts (e.g. paws, flanks/sides)				✓			
	My dog appears annoyed/upset if given less than he/she was expecting (e.g. wants table scrap and gets a pat on the head; given less food/a lower quality of food than expecting)	✓						
	My dog will attempt to escape if I try to confine him/her (e.g. in a room, crate or kennel)						✔	
	My dog is protective of his/her territory (house, garden, car)							

(✔ = suggested strong mapping of item; ✓ = potential mapping of item).

Table S5. Proposed mapping of behaviour test measures onto CFQ items for PC5.

Principal component	Item	Item mapping onto behaviour tests: Test 1 Downshift 4 treats (1a), 1 treat (1b), 0 treats (1c)	Test 2 Inability to access items (2a) Ease of removal of a range of items (2b)	Test 3 Delay in leavin g a room	Test 4 Dog ignored whilst in test room	Test 5 Ability to access food denied, restrained by lead	Test 6 Left alone in a room, experimenter out of sight (6a); in sight (6b)	Selected behaviour test measure(s) to validate principal component(s)
PC5: Frustration coping	My dog appears to cope well when denied access to things he/she is occasionally allowed (e.g. access to the sofa/bed or provision of table scraps) (R)							<u>Test 5</u><br>-Lunging frequency<br>-Vocalising (barking/whining) frequency
	I find it easy to interrupt/distract my dog from doing things he/she wants to do (R)		✓					
	My dog finds it easy to relax and settle when unable to access something he/she wants (R)		✓	✓	✓	✔		

(✔ = suggested strong mapping of item; ✓ = potential mapping of item).

Table S6. Counterbalanced test order schedule for Group 1 —'odd' group.

Test no.	Group 1 (odd) – Test	Time (mins)
	Habituation to experimenter, room and equipment<br>First saliva sample	~10
1	Downshift high value (1a) to low value (1b) to no treat (1c)	2
	Break – in test room	2
2a	Inability to access items	1
	Permitted to access items →	1
2b	Ease of removal of a range of items	1
	Break – in test room	2
3	Delay in leaving a room when lead clipped on	1
	Break – outdoors toilet break	~5
4	Dog ignored whilst in test room	5
5	Ability to access food denied, restrained by lead	2
	Break – in test rooom	2
6a	Left alone in a room, experimenter out of sight	0.5
6b	Left alone in a room, experimenter in sight	0.5
	2 minutes then second saliva sample	2
	**TOTAL**	**~37 mins**

Table S7. Counterbalanced test order schedule for Group 2 —'even' group.

Test no.	Group 2 (even) – Test order	Time (mins)
	Habituation to experimenter, room and equipment<br>First saliva sample	~10
6a	Left alone in a room, experimenter out of sight	0.5
6b	Left alone in a room, experimenter in sight	0.5
	Break – in test room	2
5	Ability to access food denied, restrained by lead	2
	Break – in test room	2
4	Dog ignored whilst in test room	5
3	Delay in leaving a room when lead clipped on	1
	Break – outdoors toilet break	~5
2a	Inability to access items	1
	Permitted to access items →	1
2b	Ease of removal of a range of items	1
	Break – in test room	2
1	Downshift high value (1a) to low value (1b) to no treat (1c)	2
	2 minutes then second saliva sample	2
	**TOTAL**	**~37 mins**

Table S8. Intraclass correlation coefficient (ICC) for inter-rater reliability assessment of behavioural measures coded from test footage.

Behavioural Measure	ICC	95% Confidence Interval		F Test with True Value 0			
		Lower Bound	Upper Bound	Value	df1	df2	Sig
Whining (frequency)	0.993	0.975	0.998	157.714	10	10	<0.001
Barking (frequency)	0.992	0.971	0.998	121.000	10	10	<0.001
Lunging (frequency)	0.928	0.734	0.980	12.973	10	10	<0.001
Ambulating (duration)	0.998	0.993	0.999	506.868	10	10	<0.001
Sitting (duration)	1.000	1.000	1.000	286291.918	10	10	<0.001
Standing (duration)	0.997	0.988	0.999	288.606	10	10	<0.001
Lying down (duration)	0.999	0.998	1.000	1780.246	10	10	<0.001

Table S9. Comparison of CFQ OQS/PCs between counterbalanced groups using Mann Whitney tests.

Canine Frustration Scale Measure	Median Score		Mann Whitney *U*	*Z*	Exact sig. (2 tailed)
	Group 1 test order 1–6 (n = 21)	Group 2 test order 6–1 (n = 23)			
OQS	0.51	0.44	185.5	−1.317	0.188
PC1	0.48	0.36	179	−1.480	0.139
PC2	0.60	0.60	239.5	−0.047	0.962
PC3	0.65	0.60	235	−0.153	0.878
PC4	0.36	0.32	159.5	−1.940	0.052
PC5	0.53	0.40	184.5	−1.358	0.174

CFQ = Canine frustration questionnaire; OQS = Overall questionnaire score; PC = principal component, where: PC1 ('General frustration'); PC2 ('Barrier frustration / perseverance'); PC3 ('Unmet expectations'); PC4 ('Autonomous control'); PC5 ('Frustration coping').

Table S10. Comparison of key behavioural measures between counterbalanced groups using Mann Whitney tests.

**Behaviour Test Measure**	**Mann Whitney *U***	***Z***	**Exact sig. (2 tailed)**
Vocalising – total number of tests	149.50	−1.120	0.269
Vocalising – total frequency across all tests	165.50	−0.663	0.512
Test 2 Lunging frequency	191.00	−0.702	0.483
Test 3 Vocalising frequency	214.50	−0.685	0.493
Test 6a/6b Scratching gate frequency	188.50	−1.853	0.064
Test 5 Vocalising frequency	206.00	−0.619	0.536

Counterbalanced groups: Group 1 test order 1–6 (n = 21); Group 2 test order 6–1 (n = 23)

Table S11. Spearman's rank order correlations between Canine Frustration Questionnaire (CFQ) principal components (PC) 2 to 5, and paired behavioural measure demonstrating convergent validity.

		Test 2 Lunging frequency	Test 3 Vocalising frequency	Test 6a/6b Scratching gate frequency	Test 5 Vocalising frequency
CFQ PC2	Corr.	**0.431***	0.286	0.130	0.208
	Sig.	**0.017**	0.06	0.402	0.182
	N	30	44	44	43
CFQ PC3	Corr.	0.263	**0.313***	−0.046	**0.429****
	Sig.	0.16	**0.038**	0.767	**0.004**
	N	30	44	44	43
CFQ PC4	Corr.	0.309	0.201	**0.376***	0.147
	Sig.	0.096	0.19	**0.012**	0.348
	N	30	44	44	43
CFQ PC5	Corr.	0.337	**0.416****	0.127	**0.447****
	Sig.	0.068	**0.005**	0.412	**0.003**
	N	30	44	44	43

(values in bold *significant at $p < 0.05$ level; **significant at $p < 0.01$ level). CFQ = Canine frustration questionnaire; PC = principal component, where: PC2 ('Barrier frustration / perseverance'); PC3 ('Unmet expectations'); PC4 ('Autonomous control'); PC5 ('Frustration coping').

Table S12. Comparison of CFQ OQS/PCs between 'behaviour test group' and 'questionnaire generation group' using Mann Whitney tests.

Canine Frustration Scale Measure	Median Score: Behaviour test group (n = 44)	Median Score: Questionnaire generation group (n = 2346)	Mann Whitney U	Z	Exact sig. (2 tailed)
OQS	0.48	0.44	42924.500	−1.916	0.055
PC1	0.36	0.36	43449.000	−1.782	0.075
PC2	**0.60**	**0.55**	**42347.000**	**−2.040**	**0.041***
PC3	**0.60**	**0.50**	**38339.000**	**−2.925**	**0.003****
PC4	0.36	0.36	48632.000	−0.646	0.518
PC5	0.47	0.47	47157.500	−0.990	0.322

(values in bold *significant at $p < 0.05$ level; **significant at $p < 0.01$ level). CFQ = Canine frustration questionnaire; OQS = Overall questionnaire score; PC = principal component, where: PC1 (‘General frustration’); PC2 (‘Barrier frustration / perseverance’); PC3 (‘Unmet expectations’); PC4 (‘Autonomous control’); PC5 (‘Frustration coping’).

Table S13. Spearman's rank order correlations between CFQ OQS/PCs, vocalising measures from behaviour test battery and cortisol levels (pre-test, post-test and pre-test to post-test change.

		Salivary cortisol levels		
		**Pre-test**	**Post-test**	**Pre-test to post-test change**
CFQ OQS	Corr.	0.132	0.296	0.280
	Sig.	0.560	0.180	0.207
	N	22	22	22
CFQ PC1	Corr.	0.143	−0.053	0.019
	Sig.	0.524	0.816	0.932
	N	22	22	22
CFQ PC2	Corr.	0.384	0.182	0.075
	Sig.	0.078	0.418	0.739
	N	22	22	22
CFQ PC3	Corr.	0.069	0.383	0.329
	Sig.	0.759	0.078	0.135
	N	22	22	22
CFQ PC4	Corr.	−0.007	0.346	0.319
	Sig.	0.975	0.115	0.148
	N	22	22	22
CFQ PC5	Corr.	−0.156	**0.477***	**0.525***
	Sig.	0.489	**0.025**	**0.012**
	N	22	22	22
Vocalising – total number of tests	Corr.	0.080	0.394	0.373
	Sig.	0.730	0.077	0.095
	N	21	21	21
Vocalising – total frequency across all tests	Corr.	0.113	0.360	0.377
	Sig.	0.625	0.109	0.092
	N	21	21	21

(values in bold *significant at $p < 0.05$ level). CFQ = Canine frustration questionnaire; OQS = Overall questionnaire score; PC = principal component, where: PC1 ('General frustration'); PC2 ('Barrier frustration / perseverance'); PC3 ('Unmet expectations'); PC4 ('Autonomous control'); PC5 ('Frustration coping').
